# The prevalence of depression symptoms among infertile women: a systematic review and meta-analysis

**DOI:** 10.1186/s40738-021-00098-3

**Published:** 2021-03-04

**Authors:** Zahra Kiani, Masoumeh Simbar, Sepideh Hajian, Farid Zayeri

**Affiliations:** 1 Student Research Committee, Department of Midwifery and Reproductive Health, School of Nursing and Midwifery, Shahid Beheshti University of Medical Sciences, Tehran, Iran; 2 Midwifery and Reproductive Health Research Center, Department of Midwifery and Reproductive Health, School of Nursing and Midwifery, Shahid Beheshti University of Medical Sciences, Tehran, Iran; 3 Proteomics Research Center and Department of Biostatistics, Faculty of Allied Medical Sciences, Shahid Beheshti University of Medical Sciences, Tehran, Iran

**Keywords:** Infertility, Women, Depression, Meta-analysis, Prevalence

## Abstract

**Background:**

Infertile women’s mental health problems, including depression, are key fertility health issues that affect infertile women more severely than infertile men. Depression may threaten the health of individuals and reduce the quality of their lives. Considering the role and impact of depression on responses to infertility treatments, a systematic review and meta-analysis were conducted to investigate the prevalence of depression symptoms among infertile women.

**Methods:**

International databases (PubMed, Cochrane Library, Web of Sciences, Scopus, Embase, and PsycINFO), national databases (SID and Magiran), and Google Scholar were searched by two independent reviewers for articles published from 2000 to April 5, 2020. The search procedure was performed in both Persian and English using keywords such as “depression,” “disorders,” “infertility,” “prevalence,” and “epidemiology.” The articles were evaluated in terms of their titles, abstracts, and full texts. The reviewers evaluated the quality of the articles using the Newcastle–Ottawa Scale, after which they analyzed the findings using STATA version 14. The I^2^ and Egger’s tests were performed to examine heterogeneity and publication bias, respectively.

**Results:**

Thirty-two articles were subjected to the meta-analysis, and a random effects model was used in the examination given the heterogeneity of the articles. The samples in the reviewed studies encompassed a total of 9679 infertile women. The lowest and highest pooled prevalence rates were 21.01% (95% confidence interval [CI]: 15.61–34.42), as determined using the Hospital Anxiety and Depression Scale, and 52.21% (95% CI: 43.51–60.91), as ascertained using the Beck Depression Inventory, respectively. The pooled prevalence values of depression among infertile women were 44.32% (95% CI: 35.65–52.99) in low- and middle-income countries and 28.03% (95% CI: 19.61–36.44) in high-income countries.

**Conclusion:**

The prevalence of depression among infertile women was higher than that among the general population of a given country. Especially in low- and middle-income countries, appropriate measures, planning, and policy that target the negative effects of depression on infertile women’s lives should be established to reduce related problems.

**Supplementary Information:**

The online version contains supplementary material available at 10.1186/s40738-021-00098-3.

## Background

Infertility is defined as the inability to achieve pregnancy after one year of unprotected intercourse [1]. This condition affects about 10 to 12% of couples worldwide [2], but the percentage is higher (about 31.1%) in low- and middle-income countries [3]. Among women, 1.9% experience primary infertility, and 10.5% grapple with secondary infertility [4]. Infertility is caused by many factors, ranging from female- and male-related determinants separately to a combination of factors; sometimes, the condition is prompted by no cause [5].

Being one of the main problems in reproductive health, infertility is a matter of serious concern for the World Health Organization (WHO). The organization stated that the disregard of infertility in different countries poses widespread psychological problems at individual and social levels [6]. Unfortunately, many nations pay insufficient attention to this condition, thereby resulting in devastating effects that not only prevent individuals from having children but also diminish their health and quality of life [7].

In many developing and developed societies, a woman is considered a complete individual only when she becomes a mother [8]. This perspective leads to inequality between men and women as well as gender discrimination [9]. Additionally, the majority of reproduction medications and treatments are performed on women, causing them considerable discomfort, feelings of sickness, and disability [10]. Many women’s infertility is also accompanied with extensive psychological changes, such as depression [11], which is followed by social isolation and low self-esteem [12], although a number of women cope with infertility and have positive and meaningful lives [13]. The thoughts that infertile women entertain about pregnancy may give rise to depression, anxiety, and stress in such a way that depression levels resemble those experienced by women with cancer [14]. These problems inevitably affect their quality of life, with some afflicted females even contemplating suicide and death given widespread social pressures in addition to depression [15].

Despite the high prevalence of infertility, however, most women do not share their problems with their families and friends, thus precluding the acquisition of social support [16]. Infertility as well as the resultant social stigma [9], fear of loneliness in the future [17], fear of divorce [18], and unpredictable treatment processes are critical factors in the development of depression among this group of women [19]. Other issues that are contributory to depression are frequent visits to doctors and medications. All these factors, in turn, affect the responses of women to infertility treatments, their health statuses, and their quality of life [20, 21]. Another issue that needs consideration is the fact that the prevalence of depression in infertile couples differs by country [22]. Nevertheless, most studies reported that infertile women experience higher levels of depression than do infertile men [23]. In Naab et al.’s [24] study, for example, depression prevails at a rate of 11%, but in the works of Crawford et al. [25], Pinar et al. [26], and Haririan et al. [27], prevalence occurs at percentages of 41, 65, and 85%, respectively.

The above-mentioned studies provided valuable insights, but no study has been devoted specifically to a meta-analysis of the prevalence of depression among infertile women. Because the sample sizes in some studies are small, the findings that they derive cannot be used as basis for decision making and policymaking at the macro level [28]. This deficiency can be addressed by supplementing research with meta-analyses as these examinations are primary sources of credible evidence for medical staff, physicians, and policymakers. Correspondingly, the present study conducted a systematic review and a meta-analysis to examine the prevalence of depression symptoms among females afflicted with infertility.

## Methods

### Search strategy

The results of this work are reported on the basis of the Preferred Reporting Items for Systematic Reviews and Meta-Analyses (PRISMA). International databases (PubMed, Cochrane Library, Web of Science, Scopus, Embase, and PsycINFO), national databases (SID and Magiran), and Google Scholar were searched by two independent reviewers for articles published from 2000 to April 5, 2020. The search procedure was performed in both Persian and English using keywords such as “depression,” “disorders,” “infertility,” “prevalence,” and “epidemiology.” The keywords were combined using AND and OR operators (Additional file 1: Search Strategy).

### Inclusion and exclusion criteria

The inclusion criteria were as follows:

■ Cross-sectional studies and longitudinal studies using cross-sectional data

■ Research that used valid methods of assessing depression symptoms (clinical interviews or standard questionnaires)

■ Works with infertile women (those with no pregnancy after one year of unprotected intercourse) and women of reproductive ages (15–49 years) as subjects

■ Studies with a minimum sample of 30 participants

■ Those involving women with no chronic diseases and cancer

The following studies were excluded from the analysis:

■ Case studies

■ Review and animal studies

■ Research on mental syndromes

■ Studies published in languages other than English and Persian

■ Original articles to which access could not be obtained

■ Unrelated reports

### Data extraction

The two reviewers separately evaluated article titles and abstracts on the basis of the inclusion criteria to determine which studies could be included in the study. Then, the full texts of prospective articles were reviewed and included in the sample if they satisfied the criteria. The required data were extracted by the trained reviewers independently, and cases of inconsistency and disagreement were resolved by a third reviewer. The information required for the assessment of each article were the names of authors, year of publication, research context (location), sample size, type of tools, type and duration of infertility, average age of women participants, and rate of depression prevalence among infertile women.

### Quality evaluation

The Newcastle–Ottawa Scale (NOS) modified by Zhang et al. [29] was used to assess the quality of the nonrandomized studies subjected to the meta-analysis. The quality of the present research was evaluated using the modified version of the NOS by Zhang et al. [27], which addresses five domains: the representativeness of a sample (Population contained a mixture of specialties at multiple sites or a single specialty at a single site), sample size (200 and greater than 200 participants or less than 200), non-respondents (Comparability between respondent and non-respondent characteristics was established, and the response rate was satisfactory), the ascertainment of anxiety (Validated measurement tool using a validated cutoff score or clinical interview), and the quality of descriptive statistics reporting Reported descriptive statistics to describe the population (e.g., age, sex) with proper measures of anxiety (e.g., standard deviation, standard error, range, percentage). The items in each section are scored from 0 to 1; hence, the minimum and maximum scores that can be obtained are 0 and 5, respectively. The studies were classified into those involving low- and high-risk groups on the basis of their NOS scores (≤3 and > 3, respectively) [27]. As with the data extraction, the quality of the articles was evaluated by the two independent reviewers, and inconsistency and disagreement were resolved by a third reviewer. The results of the quality evaluation are presented in Additional file 2. The coefficient of agreement between the reviewers was K = 0.85.

### Outcome measures

The main outcome of interest in the current research was the presence of depression symptoms among the infertile women. In the studies examined, standard instruments, namely, interviews and questionnaires, were administered to the participants to determine symptoms and identify the prevalence of depression among this population.

### Statistical analysis

The I^2^ test was carried out to assess heterogeneity, which manifests in three forms: low (25%), moderate (50%), and high (75%) heterogeneity. A random effects model was adopted in cases wherein heterogeneity was > 50% [30]. The causes of heterogeneity were explored on the grounds of meta-regression, duration of infertility, and sample size. Egger’s test was run to evaluate publication bias, with subgroup analysis performed on the basis of the types of tools used in the studies and the World Bank’s classification of countries by income. The collected data were analyzed using STATA version 14. A *P*-value of 0.05 was considered indicative of statistical significance.

## Results

**Fig. 1:**
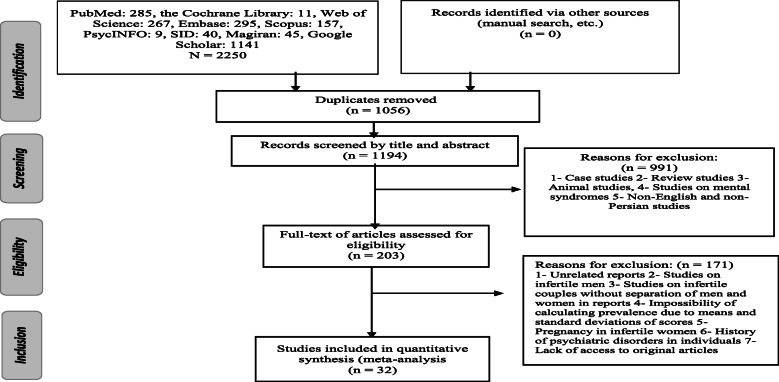
Flowchart of the selection of studies

Figure 1 shows details regarding the article selection. As can be observed, 2250 articles were found in the initial search. After duplicates were excluded, the titles and abstracts of the remaining articles were reviewed. Finally, the full texts of 203 papers were examined, after which a final sample of 32 studies were subjected to the meta-analysis.

One study each was conducted in Sweden, India, Iraq, Vietnam, Italy, Poland, Turkey, Malaysia, Tunisia, Pakistan, Saudi Arabia, and Hungary; two each were carried out in Norway, Ghana, and the US; three were performed in Nigeria; four were conducted in China; and seven studies were carried out in Iran. Among the studies, 23 studies were carried out in low- and middle-income countries, whereas nine were performed in high-income nations. To determine depression symptoms, the researchers used different instruments, such as the Mini International Neuropsychiatric Interview (MINI) (one study), International Classification of Diseases-10th Revision (ICD-10) (one study), National Institutes of Health Patient Reported Outcomes Measurement (NIH-PROMS) (one study), Depression Anxiety Stress Scales (two studies), Patient Health Questionnaire (two studies), the 10-item Center of Epidemiologic Studies Short Depression Scale (CES-D10) (two studies), Zung Depression Scale (ZDS) (four studies), Hospital Anxiety and Depression Scale (HADS) (seven studies), and Beck Depression Inventory (BDI) (12 studies).

Overall, the sample sizes used in the studies amounted to 9679 infertile women, with the minimum and maximum samples being 30 and 1413 participants, respectively. The prevalence of depression in the studies ranged from 6.4% (Norway) to 85% (Iran). Table 1 presents the information extracted from the examined studies.

**Table 1:** Characteristics of the studies selected for the meta-analysis

ID	Authors	Year publication	countries	Income levels	Sample size	Type of infertility	Prevalence of depression	Age (Y) (mean ± SD)	Mean years of infertility (mean ± SD)	Type of Tools	Quality assessment
1	Ramezanzadeh et al. [20]	2004	Iran	Low and middle	370	Primary and secondary	40.80%	28 ± 5.37	6.36 ± 4.18	BDI	3/5
2	Alosaimi et al. [31]	2015	Saudi Arabia	High	206	Primary and secondary	26.20%	NA	5.4 ± 4.3	MINI	3/5
3	Maroufizadeh et al. [2]	2018	Iran	Low and middle	649	Primary and secondary	33.90%	31/37 ± 5.69	5.62 ± 4.03	HADS	4/5
4	Joelsson et al. [32]	2017	Sweden	High	468	Primary and secondary	15.70%	30.1 ± 4.8	1.8 ± 002	HADS	5/5
5	Lakatos et al. [33]	2017	Hungary	High	134	Primary and secondary	44.80%	33.30 ± 4.85	3.61 ± 3.08	BDI	3/5
6	Yusuf [34]	2006	Pakistan	Low and middle	100	Primary and secondary	79%	24.60 ± 5.40	NA	DASS	3/5
7	Biringer et al. [35]	2015	Norway	High	615	Primary and secondary	8.20%	35.15 ± 6.28	NA	HADS	5/5
8	Upkong and Orgi [36]	2006	Nigeria	Low and middle	112	Primary and secondary	42.90%	34.5 ± 5.5	4.46 ± 3.73	BDI	4/5
9	Rostad et al. [37]	2014	Norway	High	1413	Primary and secondary	6.40%	NA	NA	HADS	3/5
10	Verma et al. [38]	2015	India	Low and middle	140	Primary and secondary	56.40%	28.70 ± 5.94	NA	HADS	3/5
11	Kalkhoran et al. [39]	2011	Iran	Low and middle	30	Primary and secondary	46.70%	29.2 0 ± 4.2	4.8 ± 0.5	BDI	3/5
12	Peyvandi et al. [40]	2009	Iran	Low and middle	200	Primary and secondary	62.00%	33.39 ± 0.2	4.1 ± 0.6	BDI	4/5
13	Sulyman et al. [41]	2019		Nigeria	207	Primary and secondary	25.60%	29.60 ± 4.0	5.60 ± 2.1	HADS	4/5
14	Vo et al. [42]	2019	Vietnam	Low and middle	401	Primary and secondary	12.20%	30.41 ± 4.47	3.10 ± 2.20	PHQ-9	4/5
15	Oladeji and OlaOlorun [43]	2018	Nigeria	Low and middle	110	Primary and secondary	52.70%	34.50 ± 5.70	NA	PHQ-9	3/5
16	Crawford et al. [25]	2017	USA	High	416	Primary and secondary	41.00%	34.25 ± 4.20	2.50 ± 0.60	NIH-PROMIS	4/5
17	Al-Asadi and Hussein [44]	2015	Iraq	Low and middle	251	Primary and secondary	68.90%	24.26 ± 8.5	NA	ICD-10	4/5
18	Alhassan et al. [45]	2014	Ghana	Low and middle	100	Primary and secondary	62.00%	30.50 ± 6.30	NA	BDI	3/5
19	Haririan eta l [27]	2009	Iran	Low and middle	100	Primary and secondary	85.00%	27.50 ± 4.40	7.01 ± 4.45	BDI	3/5
20	Chiaffarino et al. [46]	2011	Italy	High	872	Primary and secondary	17.90%	35.25 ± 6.70	NA	ZDS	4/5
21	Drosdzol and Skrzypulec [47]	2009	Poland	High	206	Primary and secondary	35.44%	29.80 ± 4.10	NA	BDI	4/5
22	Ma et al. [48]	2018	*China*	Low and middle	98	Primary and secondary	47.00%	31.80 ± 4.80	NA	ZDS	3/5
23	Wu et al. [49]	2014	China	Low and middle	288	Primary and secondary	22.60%	33.30 ± 3.80	NA	CES-D10	3/5
24	Naab et al. [24]	2013	Ghana	Low and middle	203	Primary and secondary	11.00%	31.25 ± 4.62	3.40 ± 2.70	CES-D10	4/5
25	Kissi et al. [22]	2013	Tunisia	Low and middle	100	Primary and secondary	33.00%	32.69 ± 4.91	5.19 ± 4.62	HADS	3/5
326	Pinar et al. [26]	2012	Turkey	Low and middle	160	Primary and secondary	65.00%	25.23 ± 3.25	5.23 ± 3.42	BDI	3/5
27	Psaros et al. [50]	2012	USA	High	104	Primary and secondary	63.00%	35.87 ± 0.4	NA	BDI	3/5
28	Li et al. [51]	2016	China	Low and middle	211	Primary and secondary	50.71%	NA	2.80 ± 1.20	ZDS	4/5
29	Khademi et al. [52]	2004	Iran	Low and middle	681	Primary and secondary	40.10%	27.78 ± 5.32	6.28 ± 3.83	BDI	4/5
30	Jin et al. [53]	2019	China	Low and middle	460	Primary and secondary	14.80%	29.12 ± 4.36	5.60 ± 3.20	ZDS	3/5
31	Khademi et al. [54]	2005	Iran	Low and middle	251	Primary and secondary	39.00%	29.60 ± 5.7	7.40 ± 4.6	BDI	4/5
32	Musa et al. [55]	2014	Malaysia	Low and middle	123	Primary and secondary	31.70%	NA	NA	DASS	4/5

### Evaluation of heterogeneity and meta-analysis

**Fig. 2:**
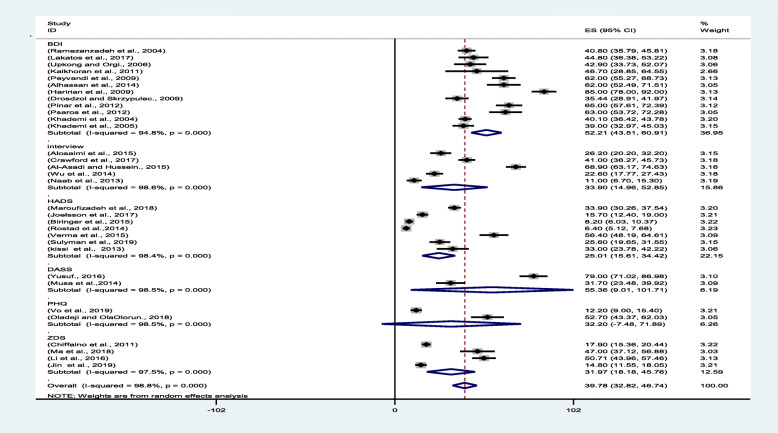
Forest plot of the pooled prevalence of depression among infertile women

The results of the I^2^ test revealed the presence of heterogeneity in the studies (I^2^ = 97.92), hence requiring the use of the random effects model. With regard to the considerable variety of instruments used to measure depression in the infertile women, a subgroup analysis was run to investigate the prevalence of the condition in this population. The studies using MINI, ICD-10, NIH-PROMS, and CES-D10 were classified under the interview subgroup, and the studies wherein other instruments were employed were also categorized in their own subgroups. Accordingly, six subgroups were defined, whose analysis results revealed that the lowest pooled prevalence rate of depression was 21.01% (95% confidence interval [CI]: 15.61–34.42), as determined using the HADS, and that the highest pooled prevalence rate of depression was 52.21% (95% CI: 43.51–60.91), as ascertained using the BDI. Furthermore, the lowest prevalence rate was 6.4% (95% CI: 5.12–7.68), derived in Rostad et al.’s study [35] in Norway, whereas the highest prevalence rate was 85.00% (95% CI: 78.00–92.00), reported in Haririan’s study [25] in Iran (Fig. 2).

### Publication Bias

**Fig. 3:**
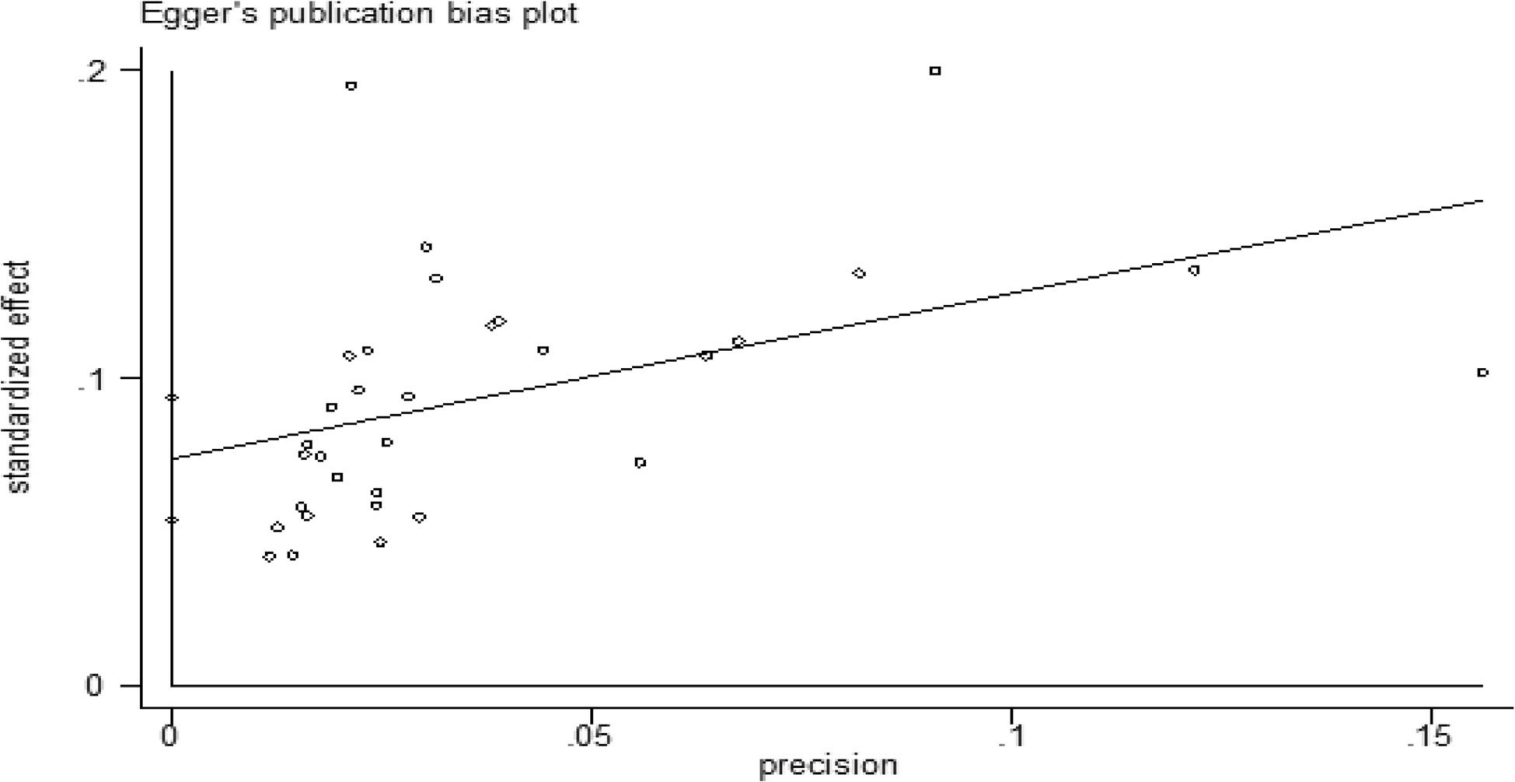
Egger’s tests for publication bias plot

The results of Egger’s tests for prevalence were statistically significant (*p* = 0.001). It means for prevalence, the results of the Egger’s test indicating that there is publication bias (Fig. 3).

### Meta-regression

Two variables were included in a univariate meta-regression as covariances to investigate the causes of heterogeneity. The results indicated that sample size (*P* = 0.342) and duration of infertility (*P* = 0.542) were the main reasons for the heterogeneity in the prevalence of depression among the infertile women.

### Subgroup analysis

**Fig. 4:**
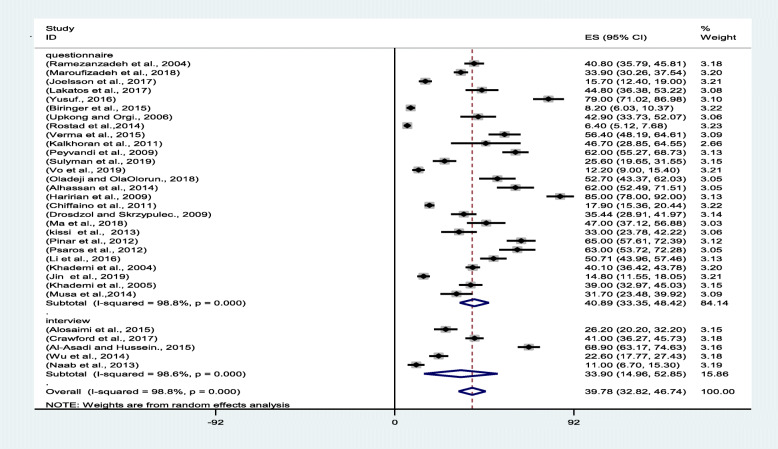
Forest plot of the pooled prevalence of depression among infertile women (interview and questionnaire subgroups)

A review of the articles indicated a lower prevalence of depression in the studies that administered clinical interviews than in the research that employed questionnaires. The subgroup analysis performed to compare these studies showed that the interview-studies found a pooled prevalence of 33.90% (95% CI: 14.961–52.85), whereas the questionnaire-based studies found such prevalence to be 40.39% (95% CI: 33.35–48.42) (Fig. 4).

**Fig. 5:**
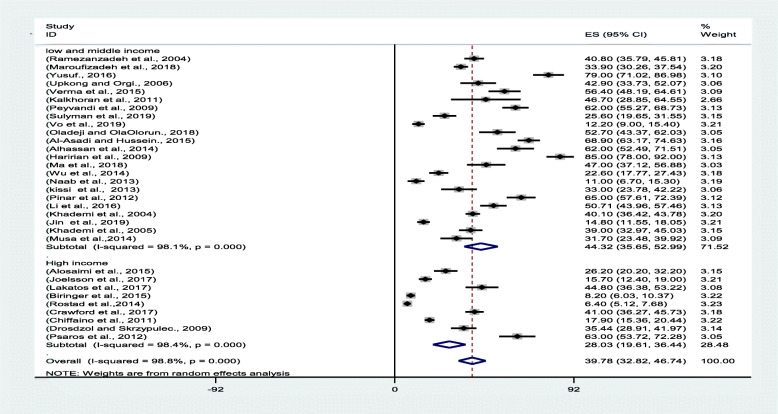
Forest plot of the pooled prevalence of depression among infertile women (income level of different countries)

The review of the texts reflected that the prevalence of depression among the infertile women was higher in low- and middle-income countries than in high-income countries. The World Bank divides countries into these two subgroups. The results indicated that the pooled prevalence of depression among the infertile women in low- and middle-income countries was 44.32% (95% CI: 35.65–52.99), whereas that of the infertile women in high-income countries was 28.03% (95% CI: 19.61–36.44) (Fig. 5).

## Discussion

This study investigated the prevalence of depression among infertile women via a systematic review and a meta-analysis. The highest pooled prevalence of depression was 52.21% (95% CI: 43.51–60.91) and was detected using the BDI, whereas the lowest was 21.01% (95% CI: 15.61–34.42) and was found using the HADS. Prevalence in the infertile women was higher than that in the general population. The WHO estimated the global prevalence of depression at 4.4% [31]. In a systematic study on the global prevalence of common mental disorders from 1980 to 2013, the pooled prevalence of these conditions was 29.2% (95% CI, 25.9–32.6%) [32]. Depression is one of the most common problems to which individuals are exposed as they encounter life’s difficulties and illnesses. This disorder may cause disability and reduce quality of life among individuals [33]. In most parts of the world, childbearing is equivalent to femininity so that women feel extreme sadness and failure in life when they fail to become mothers; their inability to conceive then triggers depression [34]. In comparison with the general population, infertile women face many social and family problems that are brought on by their condition, such as high treatment costs, social stigma, frequent doctor visits, and many medications and tests; these factors also contribute to depression [35].

The lowest and highest prevalence rates indicated that depression is more widespread among infertile women than in pregnant and postpartum women. In a systematic review, Sawyer et al. [36] showed that the prevalence of depression in pregnant women ranged from 4.3 to 17.4%. A 2018 systematic review and meta-analysis reflected that the prevalence of depression in postpartum women was 17% (95% CI 0.15–0.20) [37]. Childbearing and parenting are the important roles of women in many societies [38]; hence, their inability to conceive translates to a failure to achieve one of their main goals in life. This realization may lead to depression [39].

The present study uncovered a prevalence of 39.78% (95% CI: 32.82–46.74) among infertile women. In Yang et al.’s [40] study, the prevalence of depression among infertile men was 20.8% [56], but the WHO reported prevalence rates of 5.1 and 3.6% among women and men worldwide [31]. Women are confronted with many problems as they deal with infertility, and these problems play a role in the development of depression [19]. Some of these problems are caused by fear of the future, fear of experiencing divorce, loneliness, and rejection by others [38].

The studies subjected to this meta-analysis reported prevalence levels that ranged from 6.4 to 85%. For instance, Chiaffarino et al. [41] and Joelsson et al. [42] reported prevalence rates of 17.9 and 15.7% in Italy and Sweden, respectively, while Al-Asadi and Hussein [43] and Haririan et al. [27] discovered prevalence rates of 68.90 and 85% in Iraq and Iran, respectively. The inconsistencies in the findings seem to have been caused by the variances in income levels across the countries. On this basis, the subgroup analysis was performed with country income as reference. The results, which were grounded in the World Bank’s classification of countries, indicated prevalence levels of 44.32% among the infertile women in low- and middle-income countries (95% CI: 35.65–52.99) and 28.03% (95% CI: 19.61–36.44) among such a population in high-income countries. Depression is the seventh leading cause of disease burden in low- and middle-income countries [19] because in such contexts, health care and appropriate tools for screening individuals in terms of mental health are insufficient [44]. These problems are exacerbated by the fact that treatment costs are paid out of pocket and expenses related to health care are excessive [45]. Depression may also stem from the economic particularities of the majority of low- and middle-income countries, where women are unemployed, and their husbands pay for infertility treatments [46]. Moreover, infertility treatments require frequent referrals and treatments, yet despite all the efforts made by individuals and treatment staff, treatment outcomes remain unpredictable [47]. In these nations, as well, infertile women receive low family and social support [46], whereas in high-income countries, emphasis is placed on prevention and mental health programs, with governments allocating funding for these purposes [48]. The WHO and the 1994 Cairo Conference on Population and Development addressed infertility as an important problem in the field of reproductive health. This condition should be of great concern for different countries, especially those belonging to the low- and middle-income brackets [49].

A meta-analysis by Frederiksen et al. [50] indicated that psychological interventions for reducing depression among infertile women would result in the greater success of infertility treatments and higher rates of pregnancy. Depression is assumed as a negative influence on mental health and, consequently, physical and social health [51]. The provision of supportive services and psychological counseling intended to help women manage and cope with infertility would effectively minimize the prevalence of depression among this group [52].

One of the limitations of this study was the adoption of various tools in evaluating the prevalence of depression among infertile women as none of the tools were developed and validated specifically for this population. Another limitation is that the study reported the means and standard deviations of the scores obtained from the depression assessment tools as these values could not be used to calculate prevalence in some of the studies.

## Conclusion

The results of the meta-analysis indicated a higher prevalence of depression among infertile women than among the general population, pregnant women, and men. Given that depression and disregard of this problem severely affect responses to treatment, resolving this issue requires further attention as well as careful planning and government involvement, especially in low-income countries.

## Supplementary Information

**Additional file 1.** Search Strategy.

**Additional file 2.** Quality Assessment.

## Data Availability

The datasets used and/or analyzed during the current study are available from the corresponding author on reasonable request.

## Abbreviations

WHO: World Health Organization
PRISMA: The preferred reporting items for systematic reviews and meta-analyses
NOS: Newcastle–Ottawa Scale
NA: Not reported
MINI: Mini International Neuropsychiatric Interview
HADS: Hospital Anxiety and Depression Scale
CI: Confidence Interval
ZDS: Zung Self-Rating Depression Scale
ICD: International Classification of Disease
BDI: Beck depression inventory
DASS: Depression Anxiety Stress Scales
PHQ: The Patient Health Questionnaire
CES-D: Center of Epidemiologic Studies Short Depression Scale
NIH-PROMIS: The National Institutes of Health Patient Reported Outcomes Measurement Information System
SD: Standard Deviation
