# Supplementary material for: The prevalence of depression symptoms among infertile women: a systematic review and meta-analysis
Source: Fertil Res Pract. 2021 Mar 4;7:6. doi: 10.1186/s40738-021-00098-3 (PMC7931512; doi:10.1186/s40738-021-00098-3)
Supplement: Supplementary file 1 — Additional file 1. Search Strategy. [file 40738_2021_98_MOESM1_ESM.doc]

**Appendix 1: Search Strategy**

(depress* or depress* disorder$ or affective disorder$ or mood disorder$ or adjustment disorder$ or affective symptom$ or dysthymi*) AND (infertility* or infertility$) AND (Prevalence or Epidemiology).
