# Supplementary material for: The prevalence of depression symptoms among infertile women: a systematic review and meta-analysis
Source: Fertil Res Pract. 2021 Mar 4;7:6. doi: 10.1186/s40738-021-00098-3 (PMC7931512; doi:10.1186/s40738-021-00098-3)
Supplement: Supplementary file 2 — Additional file 2. Quality Assessment. [file 40738_2021_98_MOESM2_ESM.doc]

**Appendix 2**: Quality Assessment

Modified Newcastle-Ottawa scoring guide.

**(1) Representativeness of the sample:**

1 point: Population contained a mixture of specialties at multiple sites.

0 points: Population contained a single specialty at a single site.

**(2) Sample size:**

1 point: Sample size was 200 and greater than 200 participants.

0 points: Sample size was less than 200 participants or a convenience sample.

**(3) Non-respondents:**

1 point: Comparability between respondent and non-respondent characteristics was established, and the response rate was satisfactory.

0 points: The response rate was unsatisfactory, the comparability between respondents and non-respondents was unsatisfactory, or there was no description of the response rate or the characteristics of the responders and the non-responders.

**(4) Ascertainment of depression:**

1 point: Validated measurement tool using a validated cutoff score or clinical interview.

0 points: Non-validated measurement tool, or validated measurement tool with non-valid cutoff score (scored as such due to its low specificity).

**(5) Quality of descriptive statistics reporting:**

1 point: Reported descriptive statistics to describe the population (*e.g.*, age, sex) with proper measures of depression (*e.g.*, standard deviation, standard error, range, precentage).

0 points: Descriptive statistics were not reported, were incomplete, or did not include proper measures of depression.

**Legend:** This scale, the scoring of which ranges from 0 to 5, assesses quality in several domains: sample representativeness and size, comparability between respondents and non-respondents, ascertainment of depressive symptoms, and statistical quality. Studies were judged to be of low risk of bias (≥3 points) or high risk of bias (<3 points).

**Total = /5**

|  | **Results of Newcastle-Ottawa Risk of Bias Assessment** | | | | | | |
| --- | --- | --- | --- | --- | --- | --- | --- |
|  | **Study ID** | **Representativeness** | **Size** | **Comparability** | **Outcome** | **Statistics** | **Total** |
| **1** | **(**[**Ramezanzadeh et al., 2004**](#_ENREF_36)**)** | **0** | **1** | **0** | **1** | **1** | **3** |
| **2** |  | **1** | **1** | **1** | **0** | **0** | **3** |
| **3** | **(**[**Maroufizadeh et al., 2018**](#_ENREF_23)**)** | **1** | **1** | **0** | **1** | **1** | **4** |
| **4** |  | **1** | **1** | **1** | **1** | **1** | **5** |
| **5** |  | **1** | **0** | **0** | **1** | **1** | **3** |
| **6** | **(Yusuf., 2016)** | **0** | **0** | **1** | **1** | **1** | **3** |
| **7** |  | **1** | **1** | **1** | **1** | **1** | **5** |
| **8** |  | **0** | **1** | **1** | **1** | **1** | **4** |
| **9** | **(Rostad et al., 2014)** | **1** | **1** | **0** | **1** | **0** | **3** |
| **10** | **(Verma et al., 2015)** | **0** | **0** | **1** | **1** | **1** | **3** |
| **11** |  | **0** | **0** | **1** | **1** | **1** | **3** |
| **12** |  | **1** | **1** | **0** | **1** | **1** | **4** |
| **13** | **(Sulyman et al., 2019)** | **0** | **1** | **1** | **1** | **1** | **4** |
| **14** | **(Vo et al., 2019)** | **0** | **1** | **1** | **1** | **1** | **4** |
| **15** | **(Oladeji and OlaOlorun., 2018)** | **0** | **0** | **1** | **1** | **1** | **3** |
| **16** | **(Crawford et al., 2017)**  **cohort**  **cohort** | **0** | **1** | **1** | **1** | **1** | **4** |
| **17** | **(Al-Asadi and Hussein., 2015)** | **0** | **1** | **1** | **1** | **1** | **4** |
| **18** | **(Alhassan et al., 2014)** | **0** | **0** | **1** | **1** | **1** | **3** |
| **19** | **(Haririan et al., 2009)** | **0** | **0** | **1** | **1** | **1** | **3** |
| **20** | **(Chiffaino et al., 2011)** | **0** | **1** | **1** | **1** | **1** | **4** |
| **21** | **(Drosdzol and Skrzypulec., 2009)** | **0** | **1** | **1** | **1** | **1** | **4** |
| **22** | **(Ma et al., 2018)** | **0** | **0** | **1** | **1** | **1** | **3** |
| **23** | **(Wu et al., 2014)** | **0** | **1** | **0** | **1** | **1** | **3** |
| **24** | **(Naab et al., 2013)** | **1** | **1** | **0** | **1** | **1** | **4** |
| **25** | **(kissi et al., 2013)** | **0** | **0** | **1** | **1** | **1** | **3** |
| **26** | **(Pinar et al., 2012)** | **0** | **1** | **0** | **1** | **1** | **3** |
| **27** | **(Psaros et al., 2012)** | **0** | **0** | **1** | **1** | **1** | **3** |
| **28** | **(Li et al., 2016)** | **1** | **1** | **0** | **1** | **1** | **4** |
| **29** | **(Khademi et al., 2004)** | **0** | **1** | **1** | **1** | **1** | **4** |
| **30** | **(Jin et al., 2019)** | **0** | **1** | **1** | **1** | **0** | **3** |
| **31** | **(Khademi et al., 2005)** | **0** | **1** | **1** | **1** | **1** | **4** |
| **32** | **(Musa et al.,2014)** | **0** | **1** | **1** | **1** | **1** | **4** |
